# Reactivation and Management of Endogenous Latent Herpesviruses in the Spaceflight Environment

**DOI:** 10.1007/s00284-026-04935-w

**Published:** 2026-05-06

**Authors:** Biying Zhang, Peijun Han, Yong Liu

**Affiliations:** 1 Department of Aerospace Hygiene, Department of Aerospace Medicine, The Fourth Military Medical University, Xi’an, 710032 Shaanxi China; 2 Key Laboratory of Aerospace Medicine of Ministry of Education, The Fourth Military Medical University, Xi’an, 710032 Shaanxi China

## Abstract

The microgravity, radiation, and high-stress environment of space present unique challenges to astronauts’ physical and mental health. In this environment, interactions between the host and pathogens are altered, thereby increasing astronauts’ risk of endogenous viral infections. Notably, viral shedding detected during spaceflight does not necessarily indicate clinically significant disease, and the distinction between molecular reactivation, productive viral replication, and symptomatic infection must be carefully considered. This review aims to ensure mission success and enhance space biocapacity and biodefence by summarizing case studies and reactivation mechanisms of endogenous latent herpesviruses, the latest prevention and control strategies, and the challenges posed by host variability and antiviral efficacy in the space environment.

## Introduction

Human exploration of the universe has coincided with increasing challenges posed by the duration of space missions and the time astronauts spend in orbit. Viruses, an essential part of the human microbiome, have posed a persistent threat to humans since the beginning of space exploration in the last century. Previous studies have pointed out that the Epstein-Barr virus (EBV) and cytomegalovirus (CMV) can be reactivated and shed during spaceflights [1, 2]. Research is increasingly highlighting the significance of viruses in spaceflight. Firstly, humans coexist with billions of microorganisms (bacteria, archaea, fungi, protozoa, viruses, or phages), and the constant exchange between the human body and the natural environment is closely tied to health and disease. Secondly, spacecraft, being closed and compact environments, house diverse microbial communities, including viruses [3]. Thirdly, the extreme space conditions, such as microgravity and cosmic radiation, often disrupt the immune balance, making astronauts susceptible to microbial infections [4]. Therefore, antimicrobial materials on spacecraft surfaces, stringent sterilization procedures, and continuous, efficient air filtration have become indispensable methods for controlling viruses in spacecraft. Pre‑flight quarantine protocols, including NASA’s Health Stabilization Program [5] and the three‑tiered isolation systems implemented by Russia (https://www.roscosmos.ru/) and China (https://www.cmse.g.ov.cn/), are routinely employed to ensure astronaut health and minimize pathogen exposure prior to International Space Station missions. These measures significantly mitigate the risk of acute and chronic viral infections. Latent viral infections, in which viruses persist in host cells in a dormant state without active replication, typically do not produce infectious virus particles, and infected astronauts may not exhibit clinical symptoms, making it challenging to use standard medical measures like inspection and quarantine for prevention [6]. Additionally, the body produces a more complex stress state under multiple stressors in the unique space environment (e.g., microgravity, cosmic radiation, circadian disruption, psychological stress from isolation and confinement, and sleep deprivation), potentially causing new infection patterns, activation, and recurrent infection.

While herpesviruses represent the most well-characterized model of latency-reactivation dynamics in the spaceflight context, other viruses also exhibit latency with potential for reactivation. For example, human immunodeficiency virus (HIV) establishes latent reservoirs in CD4^+^ T cells that can reactivate upon treatment interruption, and JC polyomavirus (JCPyV) can reactivate from kidney latency to cause progressive multifocal leukoencephalopathy in immunocompromised individuals [4, 7]. While direct evidence of their reactivation during spaceflight remains limited, these well-characterized examples illustrate how latent viruses may pose potential risks under conditions of immune dysregulation. However, the present review focuses specifically on herpesviruses due to their documented high prevalence of reactivation during spaceflight and their established clinical significance for astronaut health. To ensure astronaut safety and mission success, this review compiles information from spaceflights, ground simulation studies, and research on viral evolution during space missions. It will also cover the latest infection prevention and control strategies, aiming to unveil the “invisibility cloak” of viruses and find effective measures for their control and management.

## Latent-associated Herpesviruses

The family Herpesviridae comprises a large group of enveloped, double-stranded DNA viruses characterized by their ability to establish lifelong latent infections in their hosts. Currently, over 100 herpesvirus species have been identified across vertebrates, classified into five subfamilies. The herpesvirus virion consists of a linear double-stranded DNA genome enclosed in an icosahedral capsid, surrounded by an amorphous protein layer (tegument) and a lipid bilayer envelope containing viral glycoproteins essential for host cell entry [8]. Among these, nine species are known to infect humans, classified among three of the five herpesvirus subfamilies: Alphaherpesvirinae (herpes simplex virus type 1, HSV-1; herpes simplex virus type 2, HSV-2; and varicella-zoster virus, VZV), Betaherpesvirinae (human cytomegalovirus, HCMV; human herpesvirus 6 A/6B, HHV-6 A/6B; and human herpesvirus 7, HHV-7), and Gammaherpesvirinae (Epstein-Barr virus, EBV; and Kaposi’s sarcoma-associated herpesvirus, KSHV) [8]. It should be noted that these subfamilies also contain numerous non-human herpesvirus species.

**Fig. 1:**
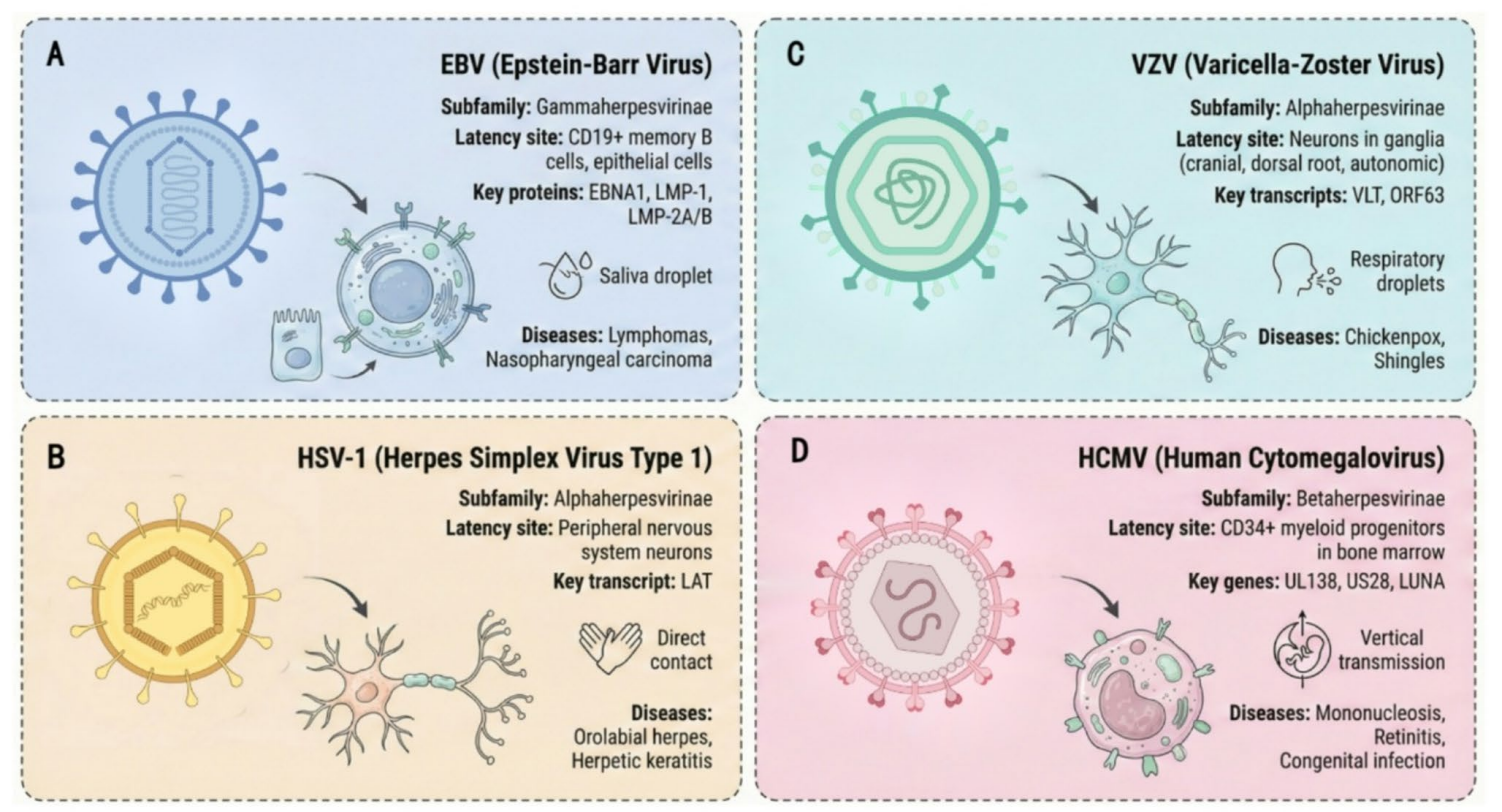
Comparative overview of four spaceflight-associated herpesviruses. (**A**) Epstein-Barr virus (EBV, Gammaherpesvirinae): establishes latency in CD19^+^ memory B cells and epithelial cells; key latent proteins include EBNA1, LMP-1, and LMP-2 A/B; transmitted via saliva droplets; associated with lymphomas and nasopharyngeal carcinoma. (**B**) Varicella-zoster virus (VZV, Alphaherpesvirinae): latent in cranial, dorsal root, and autonomic ganglia neurons; key latent transcripts include VLT and ORF63; transmitted via respiratory droplets; causes chickenpox and shingles. (**C**) Herpes simplex virus type 1 (HSV-1, Alphaherpesvirinae): latent in peripheral nervous system neurons; key latent transcript is LAT; transmitted by direct contact; causes orolabial herpes and herpetic keratitis. (**D**) Human cytomegalovirus (HCMV, Betaherpesvirinae): latent in CD34^+^ myeloid progenitors in bone marrow; key genes include UL138, US28, and LUNA; transmitted vertically; causes mononucleosis, retinitis, and congenital infection. Viral particle structures are schematic representations showing the dsDNA genome (helical structure), icosahedral capsid, tegument layer, and lipid envelope with surface glycoproteins. Arrows indicate transmission routes. Figures were created with the assistance of Figdraw (www.figdraw.com), an AI-assisted illustration platform. All content was reviewed and verified by the authors

The latent viruses that are frequently associated with space exploration primarily include EBV, VZV, HSV-1, and HCMV. The life cycle of most herpesviruses is characterized by the ability to establish latent infection, from which they can periodically reactivate to undergo lytic replication; however, alternative persistence strategies, such as low-level continuous replication or integration into the host genome, have also been described for certain family members [8]. During the lytic phase, the virus actively replicates, produces infectious virions, and typically causes cell lysis and tissue damage. In the spaceflight environment, the transition from latency to lytic reactivation is of particular concern, as the lytic phase can lead to symptomatic disease and viral shedding that may affect crew health and mission success [9]. Understanding both phases is essential for developing effective countermeasures for space missions. Each herpesvirus establishes latency in specialized host cell types; for example, the predominant latent form of EBV is found in CD19^+^ memory B cells, whereas HSV-1 and VZV are latent in neuronal cells, and latent HCMV is found in CD34^+^ myeloid progenitors. However, HCMV latent infection also exists in endothelial cells and macrophage progenitors, and EBV latent infection has been found in epithelial cells [10]. A comparative overview of four spaceflight-associated herpesviruses is presented in Fig. 1.

## EBV

EBV, a member of the human gammaherpesvirus family, infects at least 95% of the global population and is linked to approximately 200,000 cases of cancer annually [11]. EBV infection is significantly associated with several types of cancer, including Burkitt’s and Hodgkin’s lymphomas, post-transplantation lymphomas, T/NK-cell lymphomas, gastric cancer, and nasopharyngeal carcinoma. Additionally, EBV infection is associated with various non-malignant conditions, such as infectious mononucleosis, oral hairy leukoplakia, systemic lupus erythematosus, and multiple sclerosis [14]. The gene products associated with viral latency play crucial roles in EBV-induced B cell transformation, lymphomagenesis, and epithelial carcinogenesis.

The progression of EBV infection involves the following latent stages: pre-latent, latency IIb, latency III, latency IIa, and latency I (in some cases, latency 0 is also observed) [12]. EBV expresses six nuclear antigens (EBNA 1, 2, 3 A, 3B, 3 C, and EBNA-LP) and two latent membrane proteins (LMP-1 and LMP-2 A/B) at various stages in its latent genome [12]. In latent EBV infection, most viral gene promoters are methylated, associated with DNMT1, DNMT3b, and TET2 genes [13, 14]. There is an increase in EBV, such as H3K9ac, H3K27ac, and H3K4me3 [15, 16] Additionally, the higher-order chromatin structure is stably maintained with the involvement of the transcriptional regulator CTCF and cohesion subunits [12]. These compounds inhibit viral gene expression, crucial for EBV to maintain latency. EBV genes encode proteins that prevent cell apoptosis, stimulate cell proliferation, inhibit immune cell activity, reduce immune recognition molecule expression, and promote an anti-inflammatory environment [17]. These actions help the virus evade the immune response and establish persistent latent infection. After initial infection, EBV remains latent in B-lymphocytes and can reactivate periodically under specific conditions, including immunosuppression, physiological stress, hormonal changes, and co-infections, releasing infectious viral particles in saliva [18].

## VZV

VZV, belonging to the human alphaherpesvirus family, remains latent in neurons throughout the nervous system, including the cranial ganglia, dorsal root ganglia, and autonomic ganglia, following acute infection. Reactivation can cause infections and inflammatory conditions such as meningoencephalitis, cranial nerve palsies, vascular lesions, myelopathy, ocular inflammatory diseases, and skin rashes [19]. Intractable post-herpetic neuralgia (PHN) is the most well-known complication of shingles and is frequently accompanied by severe and persistent pain [19].

The molecular mechanisms underlying the latency of VZV remain primarily elusive, with the majority of current theories being extrapolated from studies on HSV-1. VZV latency-associated transcript (VLT) and ORF63 are recognized as the most critical transcripts associated with VZV latency. The polyadenylated RNA VLT, with at least five exons, is postulated to sustain the latent state during lytic infection by suppressing the transcription of ORF61 [20]. ORF63 remains in the VLT transcript as an exon, and its sole transcription is a hallmark of VZV latency [21]. Studies have shown that latent epigenetic silencing of VZV exists in the promoter euchromatin, with H3K9ac modifications of ORF63 and ORF62 [22]. Through the analysis of the genomic 3D structure and database, the researchers have identified four potential CTCF binding sites within the VZV genome [23]. This supports the hypothesis that most sites except VLT and ORF63 are bound by inhibitory chromatin to maintain VZV latency [20].

VZV reactivation can occur spontaneously or be triggered by factors such as weakened immunity due to aging, immunosuppressive therapies, illness, radiation, infection, trauma, and malignancy [21]. Primary VZV infection causes chickenpox in susceptible individuals. Immunocompetent individuals with VZV infection typically present with a blistering rash followed by flu-like symptoms [19].

## HSV-1

HSV-1, another member of the human alphaherpesvirus family, with a global prevalence rate estimated at around 67% (https://www.who.int/news/item/01-05-2020-billions-worldwide-living-with-herpes). After the initial infection of epithelial cells, the virus enters a state of latency within neurons of the peripheral nervous system. A variety of stimuli, such as fever, emotional stress, endocrine disruption, ultraviolet light exposure, trauma, and immunosuppression, have the potential to reactivate a latent viral infection. Subsequent viral shedding can facilitate the spread of the pathogen among individuals [24]. In some cases, HSV-1 can disseminate to various brain regions or affect multiple organs, including the liver, lungs, and adrenal glands, resulting in systemic, life-threatening conditions [25].

The most abundant product of HSV-1 viral gene expression during latency is the late-associated transcript (LAT). The primary LAT is rapidly spliced to produce an 8.3/9 kb primary transcript and two stable introns (2.0 and 1.5 kb) [26]. LAT inhibits the expression of ICP0 through an antisense mechanism, thereby impeding the production of infectious viral particles and facilitating the resolution of infection [27]. The amino terminus of ICP0 is crucial for the activation of the immediate-early (IE) promoter, while distinct domains govern the early (E) and late (L) promoter activation, enabling ICP0 to stimulate the expression of all viral gene classes by elevating mRNA steady-state levels [28]. In addition, latent HSV-1 produces several microRNAs (miR-H1 to miR-H8) that act in synergy with LAT to inhibit viral replication and may contribute to the inhibition of apoptosis and viral reactivation [29].

## HCMV

HCMV, a member of the human betaherpesvirus subfamily (Betaherpesvirinae), establishes and maintains latent infections similar to other characterized human herpesviruses. Among the nine human herpesviruses identified to date, the latency mechanisms of HSV-1, HSV-2, VZV, EBV, HCMV, and KSHV have been relatively well characterized, while the latency of HHV-6 A/6B and HHV-7 remains less understood. Globally, HCMV infections are prevalent, with an estimated 60% to 90% of adults harboring IgG antibodies specific to HCMV [30]. Immunocompromised individuals are particularly vulnerable to severe complications, including multi-organ inflammation and bacterial co-infections [31]. After the initial symptomless infection, HCMV enters bone marrow progenitor cells and persists for life [31]. Patients with cancer, organ transplants, and AIDS are at an exorbitantly higher risk for adverse health outcomes upon HCMV reactivation [32].

HCMV latent infection is characterized by the expression of various viral genes that maintain a stable latent state of the invading virus by influencing host cell gene expression. Latent infection of myeloid progenitor cells by HCMV is characterized by a general suppression of IE gene expression and a lack of production of infectious virus particles [33]. HCMV latency and reactivation are controlled by chromatin structure at the major immediate early promoter (MIEP) in myeloid cells. The incubation period aligns with the dominant inhibitory chromatin structure around MIEP, with HP1 and histone modifications H3K27me3 and H3K9me3 promoting HCMV latency maintenance [34]. Histone deacetylase (HDAC) activity also helps maintain chromatin inhibition [35]. Cellular transcription factors (such as YY1 and ERF) and viral factors (viral long non-coding RNA 4.9 and pp71) regulate MIEP during latency to inhibit IE gene transcription. Several studies have reported the absence of IE1/IE72 and IE2/IE86 transcripts during latency in latently infected cells when analyzing viral gene expression [33]. While defining the latent HCMV transcriptome poses challenges due to the scarce presence of naturally occurring latent cells and the complexity of the models available for study, research focusing on the virus’s transcriptional activity during latency highlights the critical role of specific viral genes in sustaining this dormant phase. These genes include UL138, US28, UL144, UL81-82ast (LUNA), and UL111A (viral IL10) [36].

## Animal Herpesviruses and Spaceflight Considerations

As human space exploration advances toward establishing permanent habitats, the potential introduction of animals for food production or companionship becomes increasingly relevant. Animal herpesviruses, such as bovine herpesvirus 1 (BHV-1), pseudorabies virus (PRV), and Marek’s disease virus (MDV) in poultry, share similar latency-reactivation mechanisms with human herpesviruses [37–39]. The stress conditions of spaceflight could potentially trigger reactivation of these viruses in animals, posing risks for food safety and animal health. Importantly, the close proximity between humans and animals in the confined spacecraft environment raises the possibility of cross-species (horizontal) transmission of herpesviruses, particularly given the immunosuppressive conditions of spaceflight. While most herpesviruses exhibit strict host specificity, certain animal herpesviruses such as PRV have demonstrated the ability to cross species barriers and cause fatal infections in non-natural hosts [38]. Therefore, future long-duration missions involving animals will require comprehensive viral screening protocols, monitoring strategies for both human and animal herpesviruses, and strict biosafety measures to prevent potential zoonotic transmission events.

## Latent Virus Reactivation-observational Study of Spaceflight

**Fig. 2:**
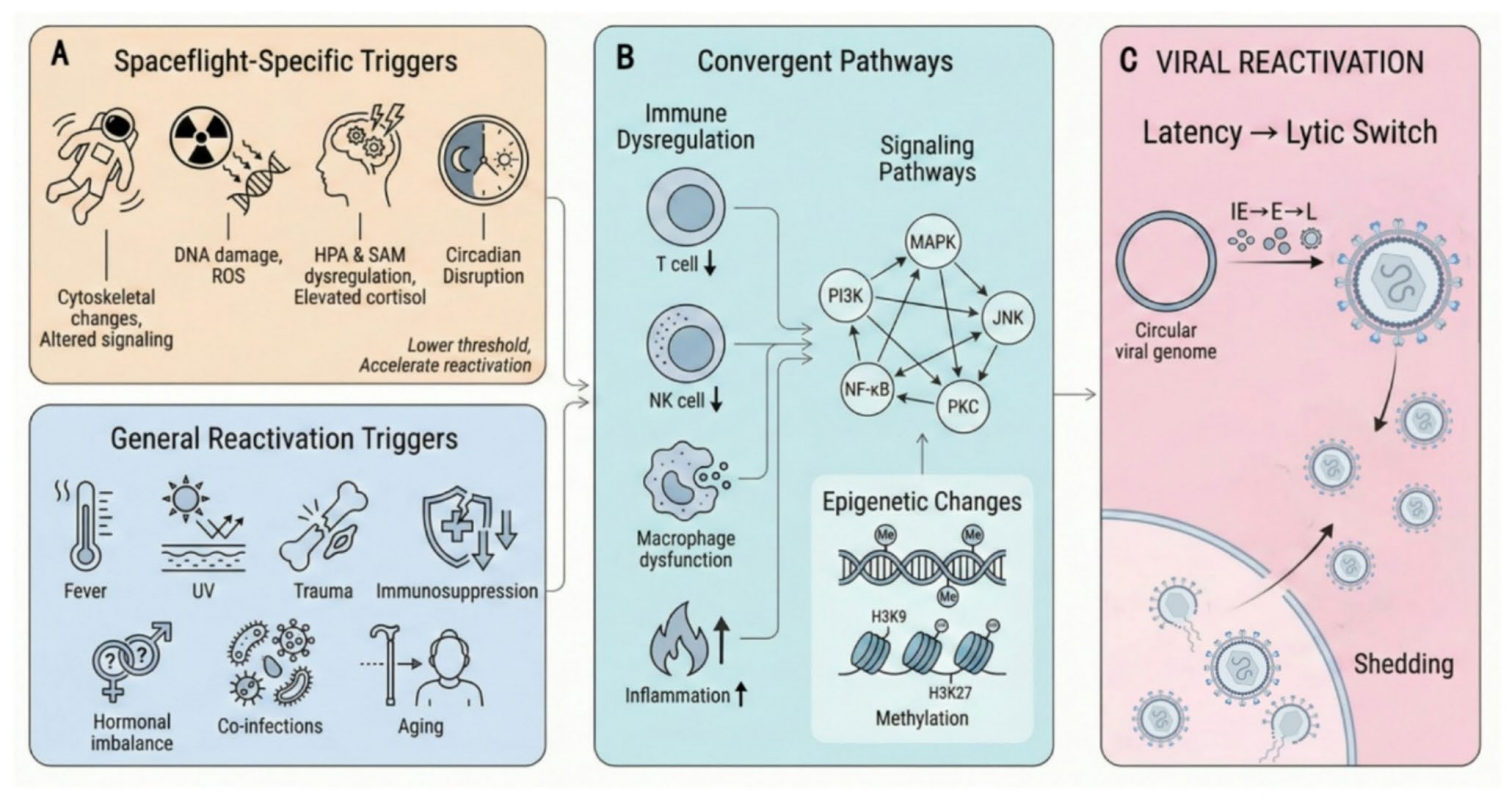
Mechanistic framework of herpesvirus reactivation in the spaceflight environment. (**A**) Spaceflight-specific triggers (upper left, yellow) include microgravity-induced cytoskeletal changes and altered signaling, cosmic radiation causing DNA damage and reactive oxygen species (ROS) generation, dysregulation of the hypothalamic-pituitary-adrenal (HPA) axis and sympathetic-adrenal-medullary (SAM) system leading to elevated cortisol, and circadian disruption. These factors lower the threshold for viral reactivation and synergize with general reactivation triggers (lower left, blue), including fever, UV exposure, trauma, immunosuppression, hormonal imbalance, co-infections, and aging. (**B**) Both categories of triggers converge on shared pathways (center, green) including immune dysregulation (decreased T cell and NK cell function, macrophage dysfunction, increased inflammation), activation of signaling cascades (MAPK, PI3K, JNK, NF-κB, PKC), and epigenetic modifications (changes in H3K9 and H3K27 methylation patterns). (**C**) These convergent pathways collectively promote the transition from viral latency to lytic replication (right, pink), involving sequential expression of immediate early (IE), early (E), and late (L) viral genes from the circular viral genome (episomal form), ultimately leading to production of infectious virions and viral shedding. Figures were created with the assistance of Figdraw (www.figdraw.com), an AI-assisted illustration platform. All content was reviewed and verified by the authors

Observational reports over the past two decades of spaceflight have shown that astronauts are at risk of latent viruses reactivating, with herpesviruses being particularly common **(**Tables 1 and 2**)**. Prolonged space missions expose astronauts to excessive environmental stress, including workload, isolation/confinement, sleep deprivation, physical exertion, noise, radiation, and microgravity. These stressors can weaken the immune system, increase stress responses, and reactivate dormant viruses, endangering astronaut health. Studies have reported an increase in VZV shedding rates from 41% during Shuttle missions to 65% during ISS missions, EBV shedding from 82% during Shuttle missions to 96% during ISS missions, and HCMV shedding from 47% during Shuttle missions to 61% during ISS missions [40]. Furthermore, a retrospective study documented a 21% HSV-1 shedding rate among ISS astronauts from 2013 to 2020, an observation that was not present in the previous five years [41]. These observations underscore the synergistic effects of multiple spaceflight stressors, including microgravity-induced immune dysregulation, cosmic radiation exposure, psychological stress from isolation and confinement, and circadian rhythm disruption, on herpesvirus reactivation **(**Fig. 2A**)**. The convergence of these factors creates a unique environment that may accelerate the transition from viral latency to lytic replication **(**Fig. 2B**)**, highlighting the need for integrated countermeasures that address multiple pathways simultaneously.

It is important to note, however, that viral shedding as detected by PCR-based assays does not necessarily equate to clinically significant disease. A substantial proportion of astronauts exhibiting viral shedding remain asymptomatic, indicating that molecular reactivation (detectable viral DNA) and productive viral replication (generation of infectious virions) are distinct from clinically manifest disease (symptomatic infection). The relationship between viral load, infectious dose, and symptom development remains poorly characterized in the spaceflight context. Therefore, while viral shedding serves as a useful biomarker for monitoring immune status and reactivation risk, it should not be used as a standalone indicator of health risk. Future studies should establish viral load thresholds that correlate with clinical outcomes and transmission risk in the confined spacecraft environment.

It should also be acknowledged that the majority of spaceflight viral reactivation data cited in this review originate from studies involving U.S. astronauts in NASA Shuttle and ISS missions. While these datasets are invaluable, spaceflight is an inherently international endeavor involving Russian cosmonauts, Chinese taikonauts, and crew members from other nations. Data from the Russian space program, including long-duration Mir station missions, have occasionally been referenced, but comprehensive viral reactivation studies from Russian or Chinese missions remain limited in the English-language literature. This may reflect differences in data accessibility, publication practices, and study design priorities across space programs. Differences in mission architectures, spacecraft environments, training protocols, dietary regimens, and medical countermeasure strategies may also influence viral reactivation patterns. Future collaborative studies across international space agencies would be valuable for determining whether the observed patterns of herpesvirus reactivation and immune dysregulation are consistent across different programs and populations.

**Table 1:** Characterization of herpesviruses activated in the spaceflight environment

Latent viruses	Latent sites	Transmission	Major clinical diseases	Protective measures	References
EBV	Lymphatic tissue	Saliva	malignant lymphoid, epithelial cancers	vaccinations	[18]
VZV	Neurons	Herpes secretions	varicella, herpes zoster	vaccinations, valacyclovir, acyclovir, famciclovir	[42]
HSV-1	Neurons	Saliva, secretions	orolabial herpes, herpetic sycosis, herpes gladiatorum	valacyclovir, acyclovir, famciclovir	[43]
HCMV	Myeloid lineage cells	Vertical transmission	infectious mononucleosis, retinitis, pneumonia	ganciclovir, cidofovir, foscarnet	[44]

**Table 2:** Case reports of herpesvirus reactivation on space missions

Viruses	Samples	Main findings	Mission hours	Years
EBV	Saliva	The mean EBV copy number was significantly higher during the flight[45]	5–14 days	2005
	Saliva	EBV was reactivated before, during, and after the flight[46]	3 days	2007
	Peripheral blood	Latent, immediate, and early viral transcripts were detected in 6 (6/6) astronauts; Latent, immediate, and early viral transcripts were detected in 6 (6/6) astronauts, with new onset of expression of late replicating transcripts after return[47]	11 days 180 days	2011
	Saliva	5 (5/17) astronauts had detectable viral shedding at 0–3 h after landing[48]	14–17 days	2013
	Saliva, peripheral blood	14 (14/17) astronauts shed EBV in saliva during all three phases of flight; EBV DNA copy numbers were elevated during the flight[49]	12–16 days	2014
	Saliva, peripheral blood	A case report of reactivation of EBV in an astronaut with persistent rash and rhinitis[50]	191 days	2016
	Saliva, peripheral blood	22 (22/23) astronauts had higher EBV copy numbers in saliva during the flight; No significant differences in EBV DNA levels in PBMC were found[51]	23 days	2017
	Saliva, peripheral blood	8 (8/8) astronauts tested positive for EBV DNA serology[52]	> 180 days	2020
VZV	Saliva	VZV was reactivated during and after the flight[46]	3 days	2007
	Saliva	Infectious VZV particles were detected in saliva in 2 (2/3) astronauts[53]	13 days	2008
	Saliva	8 (8/17) astronauts had detectable viral shedding at 0–3 h after landing[49]	14–17 days	2013
	Saliva, peripheral blood	7 (7/17) astronauts shed VZV in saliva samples both during and after flight; VZV DNA copy numbers were elevated during the flight[49]	12–16 days	2014
	Saliva, peripheral blood	A case report of reactivation of VZV in an astronaut with persistent rash and rhinitis[50]	191 days	2016
	Saliva	15 (15/23) astronauts were infected with VZV, with reactivation being the most pronounced in the later stages of the flight[51]	23 days	2017
	Saliva	Significant reactivation of VZV in 4 (4/9) astronauts peaked at 90 days of flight[54]	6–12 months	2019
	Peripheral blood	7 (7/8) astronauts tested positive for VZV DNA serology[52]	> 180 days	2020
HSV-1	Peripheral blood	6 (6/8) astronauts tested positive for HSV-1 DNA serology[52]	> 180 days	2020
	Saliva, diseased skin	Saliva samples and rash swabs on the first day of spaceflight showed positive PCR results; Astronauts developed rash lesions on the arms, chest, back, and neck[55]	≥ 200 days	2022
HCMV	Urine	HCMV was reactivated before and after the flight[46]	3 days	2007
	Urine	2 (2/17) astronauts had detectable HCMV shedding 3 h after landing[50]	14–17 days	2013
	Urine	8 of 14 (8/17) EBV shedders had HCMV expulsion during all three phases of the flight[49]	12–16 days	2014
	Urine	14 (14/23) astronauts had a significant increase in HCMV shedding during flight[51]	23 days	2017
	Peripheral blood	7 (7/8) astronauts tested positive for HCMV DNA serology[52]	> 180 days	2020

## Reactivation of Latent Viruses-ground-based Simulation Studies

Over the last twenty years, ground-based simulation studies have extensively examined the effects of microgravity, radiation, and various stressors. Experiments into herpesvirus reactivation across cellular, animal, and human models have produced outcomes consistent with those seen in spaceflight conditions **(**Table 3**)**. The − 6° head-down tilt bed rest protocol has proven to be a robust model for replicating the microgravity experienced by astronauts during space missions [56]. The Antarctic research station stands as one of the most remote and isolated locales on the planet. It exposes participants and researchers to stressors akin to those astronauts’ experience in space, including prolonged isolation and confinement, extreme cold, limited social interaction, disrupted circadian rhythms, and restricted access to medical care. Thus, Antarctica serves as a key terrestrial analog for studying the physiological and psychological impacts of spaceflight [57]. In this region, it is reported that all 16 subjects experienced EBV shedding in their saliva on at least one occasion, coincident with a reduction in cell-mediated immune (CMI) response and delayed-type hypersensitivity (DTH) reactions [58]. Additionally, five cases of clinical herpes zoster were documented among 204 individuals at the Antarctic Station, suggesting a significantly higher incidence rate compared to that observed in the general population [57]. The NASA Extreme Environment Mission Operations (NEEMO) program conducts spaceflight simulation missions at the Aquarius Undersea Research Laboratory (AURL), which is situated at Florida International University (FIU) and is arguably the sole operational undersea research facility of its kind. This project simulates mission loads, environmental isolation, extravehicular activities (EVAs), and spaceflight mission-associated risks [59]. Moreover, the Haughton-Mars Project (HMP), conducted at NASA’s Arctic Analogue Research Station near Devon Island in the Arctic, offers a terrain and geological features akin to those on Mars, establishing it as one of the most authentic Mars analog sites on Earth [60]. Experiments conducted here have shown that some phenotype, immune function, and stress hormone changes occurred in the HMP field participants, distinct from pre-mission baseline and post-mission recovery data. Of particular note is the rise in plasma EBV IgG levels, which may indicate increased viral reactivation [61]. The execution of in vitro cellular experiments offers a more feasible alternative to human-based trials. According to Brinley et al., radiation is the predominant inducement of EBV reactivation during spaceflight, surpassing the impacts of microgravity in isolation and in conjunction with other factors. Furthermore, Mehta et al. discovered that diverse radiation types can reactivate EBV in latently infected cells without affecting the immune response or cytokine production [62].

**Table 3:** Representative studies of herpesvirus reactivation in simulated spaceflight environments

Simulation factors	Simulation pathways	Main findings	Samples	Years
Extreme environmental stress	Winter Antarctic Research Station Project	Increased reactivation and shedding of latent EBV in scientific researchers[58]	Saliva	2000
Extreme environmental stress	HMP	5 (5/9) seropositive individuals had elevated plasma EBV IgG titers[61]	Peripheral blood	2007
Microgravity	Head-down Bed Rest Experiment	Subjects had mild reactivation of EBV and VZV[63]	Saliva, peripheral blood, urine	2007
Radiation and microgravity	Rotating wall vessel bioreactor with γ-ray	Radiation, microgravity, and combinations significantly increased the activation of EBV cleavage antigen[64]	Cells	2013
Extreme environmental stress	Winter Antarctic Research Station Project	There is an increased incidence of herpes zoster among scientific researchers, 33.3/1000 people per year[57]	Medical records	2017
Radiation	γ-ray, proton, carbon, and iron irradiation	γ-ray caused the highest rates of EBV reactivation and induced immediate early and late gene transcription of EBV DNA[62]	Cells	2018

## Reactivation Mechanisms

Sustaining the latency of herpesviruses is a complex biological feat that entails preserving the integrity of the viral genome, ensuring viral transmission, circumventing immune detection, suppressing lytic gene expression, fine-tuning the expression of cellular proteins, regulating viral gene expression, and ultimately reactivating the virus to initiate the lytic phase for infecting new hosts [65]. At the molecular level, these four herpesviruses share conserved core genes involved in DNA replication, capsid assembly, and virion maturation (approximately 40 core genes common to all herpesviruses), yet they exhibit distinct patterns of reactivation, viral shedding, and pathogenesis within human hosts. Each pathogen has distinct infection patterns and specific abilities to evade the host’s innate and adaptive immunity. This section summarizes factors associated with the reactivation of EBV, VZV, HSV-1, and HCMV **(**Table 4**)**.

While many reactivation triggers are shared between terrestrial and spaceflight environments (including psychological stress, immunosuppression, hormonal fluctuations, and co-infections; Fig. 2B), the space environment presents unique factors that may amplify or modify these mechanisms **(**Fig. 2A**)**. Specifically, microgravity-induced cytoskeletal changes can alter intracellular signaling pathways, cosmic radiation can cause direct DNA damage and oxidative stress, and the combined psychological and physiological stressors of spaceflight can chronically elevate glucocorticoid levels [9]. These spaceflight-specific factors may lower the threshold for reactivation or accelerate the kinetics of the latent-to-lytic switch, distinguishing the space environment from typical terrestrial reactivation scenarios.

Many factors, such as fever, stress, hormonal imbalance, UV exposure, trauma, and a weakened immune system, can cause the reactivation of latent herpesviruses **(**Fig. 2B**)**. Briefly, the reactivation mechanism of Epstein-Barr virus (EBV) involves the induction of cellular components and the subsequent interaction of downstream molecular signaling pathways, specifically the MAPK and PI3K pathways, with the ultimate goal of activating the BZLF1 and/or BRLF1 promoters to facilitate the lytic cycle of EBV [66]. The HCMV lysogeny’s IE gene is expressed by causing the MIEP to transition from a repressive state to an active promoter state [67]. Mehta et al. demonstrated that secondary mutations in the HSV-1 genome occur significantly in the space environment. HCMV reactivation can be triggered by allogeneic cell stimulation [55], which may partially account for the link between HCMV reactivation and graft-versus-host disease.

During extended space missions, exposure to microgravity impairs immune cell function, morphology, and differentiation. Such effects include the impairment of host immune defenses, a decline in the anti-inflammatory capabilities of monocytes/macrophages, the suppression of T cell signaling and functionality, the generation of persistent inflammation, the activation of microglia triggering neuroinflammation, and the diminished efficacy of NK cells, which play a pivotal role in immune surveillance and the response to viral infections [45, 48–50]. These immunological alterations can have profound consequences for the health and safety of astronauts. Mehta et al. found a positive correlation between plasma cytokine levels in astronauts and the reactivation of viruses, underscoring the link between immune dysregulation and viral reactivation in space [48]. The reactivation of latent herpesviruses could indicate a weakened adaptive immune response, particularly affecting the functionality of cytotoxic T lymphocytes.

As humans embark on space exploration, they encounter a myriad of environmental shifts. To tackle these stressors, the body relies on stress response systems, notably the sympathetic adrenomedullary system (SAM) and the hypothalamic-pituitary-adrenal axis (HPA). The SAM acts as a vanguard, while the HPA is a post-manipulator. During spaceflight, elevated levels of stress hormones are noted, including cortisol, dehydroepiandrosterone (DHEA), adrenaline (AD), and noradrenaline (NE) [55]. Stowe et al. have linked heightened stress hormone levels to EBV reactivation among astronauts [68]. Additionally, Mehta et al. observed an increased salivary cortisol to DHEA molar ratio in astronauts during spaceflight, indicating a possible immune system challenge [49]. However, the link to viral reactivation was not significant, likely due to a limited sample size. Alterations in the HPA and SAM axes can lead to immune dysregulation, impacting salivary antimicrobial proteins (sAMPs) and potentially causing latent viral reactivation in ISS crew members [52]. Elevated stress hormones can reactivate latent herpesviruses in astronauts due to a decline in cellular immunity.

**Table 4:** Mechanisms of reactivation of four astronaut-associated latent herpesviruses

Viruses	Relevant factors		Important players in reactivation	References
EBV	Chemical agents or inducers		Calcium ionophores, TPA, HDAC inhibitors, and sodium butyrate	[69]
	Biological inducers		DNA methyltransferases, anti-Ig, and TGF-β	[70]
	Biological entities	*A. actinomycetemcomitans*	Produces cytolethal distending toxin (CDT)	[71]
		*P. gingivalis*	Inhibits the HDAC	[72]
		*H. pylori*	The production of NH2Cl triggers the TlpD chemoreceptor	[73]
		*S. pyogenes* and *T. pallidum*	Tend to activate TLR2	[74]
	Molecular signaling pathways		Activation of the PI3K-AKT pathway	[75]
			Activation of the c-JNK-STAT pathway	[75]
			Activation of the MAPK pathway	[70]
			Activation of the PCK pathway	[76]
	Epigenetic regulation		DNA methylation: Ser^186^	[77]
			High levels of histone acetylation and H3K4me3 markers	[78]
	Cellular stresses	Oxidative stress	ROS (chemotherapy and γ irradiation)	[79]
		Hypoxia	Hypoxia-inducible factor 1 (HIF-1)	[80]
		Inflammation	COX-2 (EP receptor-signaling pathway)	[81]
VZV	Molecular signaling pathway		JNK pathway	[82]
	Epigenetic regulation		H3K4 methylation or H3K9 demethylation	[83]
	Cellular stresses	Pregnancy	Protect fetal immunity	[84]
		Lower temperature	34 °C was associated with enhanced VZV reactivation	[85]
		Ultraviolet radiation	Seasonal ultraviolet radiation enhancement and incidence of VZV	[86]
HSV-1	Molecular signaling pathways		Activation of the PI3K-AKT-mTOR pathway	[87]
			Activation of the NGF-TrkA-PI3K pathway	[88]
			Activation of the DLK/JIP-3 JNK stress pathway	[89]
	Molecular switch	Herpesvirus entry mediator (HVEM)	HSV-1 reactivation depends on HVEM immune function	[90]
	Epigenetic regulation	CTCF	The depletion drives reactivation	[91]
		HCF-1	Interactions with multiple epigenetic factors/complexes promote viral reactivation	[92]
	Cellular stresses	Ultraviolet radiation	UVB can cause latent HSV-1 reactivation	[93]
		Heat stress	Inhibits CD8 T cell function	[94]
		Stress hormone	Elevated cortisol, DHEA, AD, and NE levels	[95]
			Dexamethasone stimulation	[96]
HCMV	Biological inducers	PMA	Activation of HCMV MIE gene expression	[97]
		G-CSF, plerixafor	Reactivate HCMV during hematopoietic cell mobilization	[98]
	Chemical agents or inducers	Phorbol ester	PMA or TPA activates HCMV in THP-1	[99]
	Molecular signaling pathways		Activation of the ERK-MAPK pathway	[100]
			Activation of the PKA-CERB pathway;	[101]
			Inhibition of MEK/ERK, STAT, or PI3K/AKT pathways	[102]
	Molecular switch	Growth factor receptor (EGFR)	Inhibition of EGFR or PI3K promotes reactivation	[103]
	Cellular stresses	Inflammation	IL-6 promotes HCMV reactivation	[104]

## HSV-1 Adaptive Evolution in the Spaceflight Context

Among the four spaceflight-associated herpesviruses discussed above, HSV-1 has received particular attention in the context of adaptive evolution due to its well-characterized genome, high antigenic diversity, and documented evidence of genomic mutations during spaceflight [55]. While adaptive evolution studies of EBV, VZV, and HCMV under spaceflight conditions remain limited, HSV-1 serves as an informative model for understanding how space-specific stressors may drive viral evolution. Researchers continuously study the factors influencing viral variation, evolution, and host adaptation to develop viruses for clinical use under controlled laboratory conditions. These applications encompass the generation of avirulent strains, such as live attenuated vaccines, and the assessment of shifts in virulence and antibiotic resistance profiles utilizing virulent strains [105]. Researchers often opt to investigate the evolution of animal viruses in vitro settings due to ethical considerations and practical constraints, in contrast to the study of plant and bacterial viruses. RNA viruses generally evolve rapidly, causing acute infections, while DNA viruses mutate more slowly, leading to chronic or long-term infections [105].

The HSV-1 genome, known for its antigenic diversity, has coevolved with the human genome over a long period [106]. Searching for evidence of balanced selection could help elucidate the variations in human immune responses. Positive selection aims to link viral genotypes with virulence to develop intervention measures for clinical treatment. Investigations into the adaptive evolution of HSV have been conducted outside of aerospace settings. The property of latency reactivation is crucial for the long-term survival of herpesviruses, allowing for numerous opportunities for viral evolution within the host. A recent study has revealed that HSV-1 and Herpes simplex virus type 2 (HSV-2) generate genetic diversity de novo in different ways under the same in vitro culture conditions [107]. HSV-1 can rapidly evolve in specific environments, aided by the diversity of viral populations.

The potential for accelerated adaptive evolution of herpesviruses under spaceflight conditions warrants further investigation. Future studies employing continuous passaging under simulated microgravity (using rotating wall vessel bioreactors) and defined radiation doses, followed by whole-genome sequencing, could help identify mutations associated with virulence enhancement or attenuation. Such research would be essential for understanding the long-term risks posed by herpesvirus evolution during extended space missions. The implications of potential viral evolution for astronaut health and biosafety are significant.

## Response Strategies for Herpesvirus Reactivation

As space missions extend in duration, comprehensive strategies for managing herpesvirus reactivation become increasingly critical. These strategies can be organized into three complementary approaches: diagnostics and monitoring, preventive and therapeutic interventions, and lifestyle and nutritional countermeasures.

## Diagnostics and Monitoring of Herpesvirus Reactivation

With the extension of space mission durations, accurate and prompt diagnosis of causative microorganisms becomes more critical. Accomplishing this goal relies significantly on the development and sensitivity of detection devices.

The MinION (Oxford Nanopore Technologies) is a portable, palm-sized nanopore sequencing device that enables real-time DNA/RNA sequencing by measuring ionic current changes as nucleic acid molecules pass through biological nanopores [108]. This technology eliminates the need for PCR amplification, enabling rapid pathogen identification. Notably, nanopore sequencing has been successfully applied to herpesvirus genomic characterization. Burton et al. confirmed the potential of MinION for identifying microorganisms directly on the ISS [109], and Stahl-Rommel et al. developed a simplified swab-to-sequencer method for real-time microbiota analysis on the ISS, accessible even for crew members without specialized training [110]. These advances suggest that nanopore-based herpesvirus detection and genotyping could be feasible during spaceflight missions.

However, implementation of such technologies in space missions faces several challenges: (1) limited data transmission bandwidth for uploading large sequencing datasets to Earth-based analysis centers, particularly for missions beyond low Earth orbit; (2) requirements for device robustness, calibration, and reagent stability in microgravity environments; (3) the need for simplified, failure-resistant protocols that minimize crew training time; and (4) potential for flow cell membrane disruption due to launch vibrations and difficulties in handling air bubbles in microgravity [108].

Beyond nanopore sequencing, several other portable diagnostic technologies hold promise for spaceflight applications. Portable quantitative PCR (qPCR) devices, such as the miniPCR system that has been successfully demonstrated aboard the ISS, can provide rapid and sensitive detection of herpesvirus DNA with established protocols for EBV, VZV, HSV-1, and HCMV [108, 110]. Isothermal amplification methods, including loop-mediated isothermal amplification (LAMP) and recombinase polymerase amplification (RPA), offer advantages for space applications as they do not require thermal cycling equipment and can provide results within 30–60 min [111]. Lateral flow devices (LFDs) represent another promising approach, offering rapid point-of-care detection of viral antigens or antibodies without instrumentation. While LFDs offer simplicity and speed, their sensitivity for detecting low-level viral reactivation may be limited compared to nucleic acid-based methods. The optimal diagnostic strategy for long-duration missions may therefore combine multiple complementary platforms to balance sensitivity, specificity, speed, and operational simplicity.

Imaging techniques that operate across various scales are crucial for unraveling disease mechanisms at the organismal, tissue, and cellular levels. Andriasyan et al. developed a combination of imaging patterns and deep learning methodologies that can discern herpesvirus-infected cells without virus-specific staining, revealing discernible, virus-specific nuclear patterns applicable to related viruses within the herpesvirus family [112]. However, the transferability of such imaging-based approaches to the space environment presents significant challenges, including the need for high-resolution microscopy equipment, stable sample preparation conditions in microgravity, and substantial computational resources for deep learning inference. Miniaturized imaging systems and edge computing solutions would need to be developed before such approaches could be deployed on long-duration missions.

Using biomarkers to predict dormant virus reactivation represents a convenient and promising strategy. Urbaniak et al. pioneered research into how spaceflight affects the salivary microbiome, identifying bacterial biomarkers potentially associated with viral reactivation [113]. Their findings indicate that Gracilibacteria, Abiotrophia, Veillonella, and Haemophilus may be involved in EBV reactivation or replication. Additionally, Zhou et al. showed that HCMV miRNA profiles undergo distinct transitions from latency to reactivation, with hcmv-miR-US25-1-3p potentially serving as a predictive biomarker [114]. Estévez et al. demonstrated that specific gene expression signatures in peripheral blood could identify tuberculosis cases early, suggesting that similar immunological variables could serve as biomarkers for screening latent viral reactivations in astronauts [115].

## Preventive and Therapeutic Strategies

IFN-γ plays a crucial role in regulating chronic and latent herpesvirus infections, governing latency-reactivation dynamics. Schilling et al. demonstrated that MxB, a type I interferon-induced restriction factor, regulates infections across all three herpesvirus subfamilies, substantially reducing early gene expression of HSV-1, HSV-2, and HCMV, presenting new therapeutic possibilities for managing herpesvirus infections in space [116].

Without a universal remedy for herpesvirus infections, Asha et al. proposed that anti-inflammatory agents targeting lipid-mediated pathways, including lipoproteins, aspirin-triggered lipoproteins, resolvins, and their synthetic analogs, may serve as beneficial adjuvants to antiviral drugs and immunotherapies [117].

Although the varicella vaccine markedly diminishes incidence and mortality associated with primary VZV infections, it does not prevent neuronal latency or mitigate reactivation risk in immunocompromised individuals. Rajbhandari et al. identified Nectin-1 as a key mediator for VZV entry into neurons, offering a promising target for enhanced therapeutic and preventive strategies [118]. Future development of vaccines specifically designed to prevent reactivation, rather than primary infection, may be particularly relevant for astronaut populations.

For long-duration missions, integrating prophylactic antiviral administration with continuous immune monitoring protocols may provide an effective strategy. Such an approach would involve baseline immune profiling before launch, regular monitoring of immune biomarkers and viral shedding during missions, and triggering of prophylactic antiviral treatment when early signs of immune suppression or viral reactivation are detected.

An important consideration currently lacking in the literature is whether spaceflight-associated factors may alter antiviral drug efficacy. Microgravity has been shown to affect drug pharmacokinetics, including altered absorption due to changes in gastrointestinal motility, modified distribution resulting from fluid shifts, and potentially changed hepatic metabolism [119]. These alterations could affect intracellular drug activation, particularly for nucleoside analogs such as acyclovir and ganciclovir that require phosphorylation by both viral and cellular kinases. Furthermore, the increased mutation rates observed in microorganisms under spaceflight conditions raise the possibility that antiviral resistance mutations may emerge more readily during prolonged missions, particularly if subtherapeutic drug concentrations result from altered pharmacokinetics. Ionizing radiation may also interact with antiviral compounds, potentially affecting their stability and efficacy. Future research should systematically evaluate antiviral drug pharmacokinetics and pharmacodynamics under simulated spaceflight conditions and establish appropriate dosing guidelines for long-duration missions.

## Lifestyle and Nutritional Interventions

Herpesvirus reactivation is often associated with elevated oxidative stress, positioning antioxidant compounds as promising preventative agents. Natural food-derived compounds, including curcumin, resveratrol, and epigallocatechin gallate (EGCG), have shown efficacy against EBV-related cancers. These substances may reduce oxidative stress levels and enhance radioprotective effects on normal cells, potentially protecting astronauts from spaceflight radiation-induced viral reactivation [120].

Agha et al. assessed fitness parameters in ISS crew members and found that high pre-flight upper body muscular endurance correlated with reduced viral reactivation risk, particularly for EBV and VZV. Crew members with lower pre-flight cardiorespiratory fitness (CRF) and greater post-mission CRF deregulation exhibited the highest reactivation rates [121]. These findings suggest that optimized exercise protocols could complement pharmacological strategies for preventing viral reactivation during extended spaceflights.

Vitamin D status appears particularly relevant, as higher vitamin D levels are linked to reduced viral shedding in saliva [9]. Given that astronauts often experience vitamin D insufficiency during space missions due to limited sunlight exposure, supplementation may be warranted [122]. Additionally, Guo et al. demonstrated that adequate methionine supply and an intact methionine-folate cycle maintain EBV gene silencing, suggesting that nutritional interventions targeting one-carbon metabolism may help suppress latent virus reactivation [11].

## Conclusions and Future Directions

This review has summarized the current understanding of herpesvirus latency and reactivation mechanisms in the context of spaceflight, as well as emerging strategies for prevention and management. The unique combination of microgravity, cosmic radiation, psychological stress, and immune dysregulation in space creates an environment that significantly increases the risk of latent herpesvirus reactivation, with documented increases in viral shedding rates during both Shuttle and ISS missions. However, it is important to distinguish between viral shedding, productive replication, and clinically manifest disease when assessing the actual health risks to astronauts.

## Future Research Priorities

An additional dimension that warrants consideration is the inter-individual variability in host susceptibility to herpesvirus reactivation. Astronauts represent a relatively small and selected population, yet they are not immunologically homogeneous. Genetic polymorphisms in immune-related pathways, such as HLA alleles, killer immunoglobulin-like receptor (KIR) gene variants, and polymorphisms in cytokine genes (e.g., IL-10, TNF-α, IFN-γ), may substantially influence viral latency maintenance and reactivation risk. Epigenetic drift induced by chronic stress and cumulative radiation exposure during missions may further modulate immune gene expression over time. Sex-based differences in immune responses are also relevant, as studies have shown that women generally mount stronger antiviral immune responses than men, potentially influencing reactivation patterns in mixed-gender crews [123]. Incorporating these dimensions of host variability into risk assessment models would strengthen the relevance of countermeasure strategies and support the development of personalized approaches to herpesvirus management during spaceflight.

Several key research priorities emerge from this synthesis. First, for spaceflight applications specifically, real-time viral monitoring systems capable of detecting early signs of reactivation before symptomatic disease develops are urgently needed. Integration of portable sequencing technologies with automated sample processing could enable routine crew health monitoring during long-duration missions. Second, predictive modeling of host-virus interactions under spaceflight conditions, potentially incorporating artificial intelligence and machine learning approaches, could identify astronauts at elevated reactivation risk and enable personalized countermeasure deployment. The immune system employs multiple components to detect and respond to viral infections, including cytosolic sensors and adaptors (RIG-I-MAVS, cGAS-STING, IFI16), immune kinases and transcription factors (IKKβ-NF-κB, TBK1-IRF3), and ISGs with direct antiviral activity [124]. Identifying new pathogenic pathways or critical targets will be crucial for developing novel strategies to combat viral infections in space. Third, understanding how spaceflight-induced viral evolution and adaptation may alter virulence, transmissibility, or drug resistance profiles is critical for developing robust countermeasures. Studies have demonstrated that viral mutations are not random but follow specific constraints and are bi-directional, suggesting that higher mutation tolerance in antigenic sites may drive rapid viral evolution, while lower mutation tolerance sites represent promising vaccine targets. Fourth, the findings regarding viral receptor evolution and host adaptation have important implications specifically for designing vaccines and antiviral drugs that remain effective under the unique immunological conditions of spaceflight. Viral receptors are cell surface proteins that viruses hijack to facilitate their infection mechanisms; therefore, it is essential to investigate how the evolution of viruses may have impacted the adaptive evolution of human viral receptors to inform the development of next-generation vaccines and broadly-acting antivirals.

## Ethical and Biosafety Considerations

As viral genome manipulation tools such as CRISPR-Cas systems and bacterial artificial chromosome (BAC) platforms become increasingly accessible, their potential application in space research environments raises important ethical and biosafety considerations. The BAC system serves as a critical platform for manipulating large viral genomes, including those of herpesviruses [125]. Strict protocols must be established to prevent the inadvertent creation or release of enhanced viral variants, and international guidelines for conducting viral research in space habitats should be developed before such research becomes routine.

A particularly critical concern is the potential re-entry and dissemination of space-adapted viral strains to terrestrial populations. Viral variants that have undergone selection under spaceflight conditions (microgravity, radiation, immune-compromised hosts) may possess altered virulence, transmissibility, or drug resistance profiles that could pose novel public health risks upon return to Earth. Existing planetary protection protocols, primarily designed to prevent forward and backward contamination between Earth and other celestial bodies, may need to be expanded to address the biosafety implications of returning potentially evolved pathogens. Furthermore, current regulatory frameworks governing viral passaging and genome manipulation experiments were developed for terrestrial laboratory settings and may be insufficient for the unique challenges of space-based research. International oversight mechanisms, potentially coordinated through agencies such as the Committee on Space Research (COSPAR) and national space agencies, should be established to govern viral experimentation during long-duration missions, including mandatory risk assessments, containment protocols, and post-mission quarantine and screening procedures for both crew members and biological samples.

## Implications for Deep Space Exploration

Looking toward future lunar missions, Mars expeditions, and beyond, the challenges of herpesvirus management will be amplified by communication delays preventing real-time medical consultation with Earth, the impossibility of emergency evacuation, and mission durations extending to years rather than months. These factors underscore the need for autonomous diagnostic and treatment capabilities, comprehensive crew selection criteria that consider viral latency status and immune profiles, and robust planetary biosafety protocols.

In conclusion, a comprehensive understanding of how latent infections evolve under spaceflight conditions, coupled with the deployment of advanced diagnostic, preventive, and therapeutic technologies, is indispensable for safeguarding astronaut health and ensuring mission success. Furthermore, these efforts will enhance our understanding of human-microbe interactions in space, making it an excellent environment for developing novel therapeutics with terrestrial applications. Our ongoing efforts aim to facilitate longer stays in low-Earth orbit, support upcoming lunar missions, traverse the Van Allen radiation belts, and enable human voyages to Mars and beyond.

## Data Availability

No datasets were generated or analysed during the current study.
